# Giant angiomyofibroblastoma of the scrotum: a case report and review of the literature

**DOI:** 10.1186/s12894-025-01785-9

**Published:** 2025-05-05

**Authors:** Lianglong Zhang, Wufuer KaDee, Feng Xu

**Affiliations:** 1 Urinary Surgery General, Hami Central Hospital Affiliated to Xinjiang Medical University, N0. 11 Yizhou District, Square North Road, Hami, Xinjiang 839000 China; 2 Urinary Surgery General, The First Affiliated Hospital of Zhengzhou Medical University, Zhengzhou City, Henan Province 450000 China

**Keywords:** Angiofibroblastoma, Case report, Scrotum, Tumor

## Abstract

**Background:**

An angiomyofibroblastoma (AMF) is a rare tumor that primarily occurs in the vulva of women. AMF rarely occurs in the inguinal region and scrotum of men.

**Case presentation:**

A 59-year-old male was admitted to the hospital for evaluation of left scrotal enlargement for 2 years. A physical examination revealed no elevation in the bilateral renal or suprapubic region. The bladder was located below the pubic bone and was non- tender without pressure. Auscultation revealed no abnormalities in the right or left renal regions. The left scrotum was enlarged with a palpable mass measuring 25 × 15 cm in size. The mass was characterized by a tough, smooth surface with a clear boundary. The left testis was not palpable and the transillumination test result was negative. Magnetic resonance imaging included an abnormal signal in the scrotum, which was consistent with a space-occupying germ cell tumor but other diagnoses could not be ruled out. The preoperative preparation indicated no contraindications to surgery. Under lumbar anesthesia, the left scrotal lesion was resected. The postoperative pathologic evaluation confirmed an AMF. Currently, the patient has recovered fully without complications.

**Conclusion:**

A large-sized AMF is relatively rare in the male scrotum, but reported in this case. A scrotal AMF often has an oval shape with no palpable pain. Imaging techniques can facilitate the hypervascular status of an AMF and pathologic findings can establish the diagnosis. However, reports of scrotal AMF are limited. A more thorough understanding should be achieved with additional cases and long-term follow-up.

**Supplementary Information:**

The online version contains supplementary material available at 10.1186/s12894-025-01785-9.

## Background

An angiomyofibroblastoma (AMF) is a rare myofibroblastic tumor that primarily occurs in the vulva and genital tract of women during the reproductive years [1, 2]. In rare cases, AMF occurs in the inguinal region and scrotum of men [1, 2]. AMF is a rare, benign, soft tissue mesenchymal tumor that is named for its rich vascularity and myofibroblast-like tumor cells. Usually, the patient detects a mass or cyst with a soft boundary that is slow growing.

The pathogenesis of scrotal AMF is not fully understood. Research on the etiology, pathologic features, clinical manifestations, and treatment of scrotal AMF is limited. Scrotal AMF is often misdiagnosed as a testicular tumor. The diagnosis of AMF depends on the pathologic examination and should be differentiated from testicular malignant tumors, lipomas, cellular angiofibromas, and aggressive angiomyxomas [3]. An accurate diagnosis and effective treatment of scrotal AMF can be established through medical imaging techniques, such as ultrasound [4], computed tomography (CT) [5], and magnetic resonance imaging (MRI) [2]. Surgical resection is the primary treatment and recurrences are rare [6].

Herein we report a rare case of AMF in the scrotum with a literature review and summary of the existing research on scrotal AMF. We have provided a reliable reference for the diagnosis and treatment of scrotal AMF by deepening our understanding of the etiologic mechanism, pathologic features, and clinical manifestations through a literature review.

## Case presentation

### General information

This case report was approved by the Institutional Ethics Review Board of Hami Central Hospital affiliated to Xinjiang Medical University (LLY2024072412). The patient gave written informed consent for the study and publication of a report.

A 59-year-old male patient was admitted to the Department of Urology at the hospital with a 2-year history of left scrotal swelling, which was initially diagnosed as left testicular swelling seriously. The patient was a herdsman in a remote mountain village, with a low education level and little attention to his own health problems. He came to see the doctor because the swelling had seriously affected his own life. He denied a history of hypertension, coronary artery disease, diabetes mellitus, or other diseases. The body mass index (BMI) was 25 kg/m^2^. The physical examination revealed no elevation in the kidney area or the suprapubic region. The kidneys were not palpable and there was no costal ridge, costal lumbar, upper ureteral, quarterly costal, or middle ureteral tenderness. The bladder was located below the pubic bone and was without tenderness or pressure. Auscultation revealed no abnormalities in the right or left renal regions. The left scrotum was enlarged with a palpable mass measuring 25 × 15 cm and characterized by a tough, smooth surface and clear boundary. The left testis was not palpable and the transillumination test result was negative.

### Ancillary examinations

The ancillary examination results were as follows: human chorionic gonadotropin, 0.72 mIU/ml; alpha-fetoprotein, 4.37 ng/ml; and *Mycobacterium tuberculosis* nucleic acid test, negative.

**Fig. 1:**
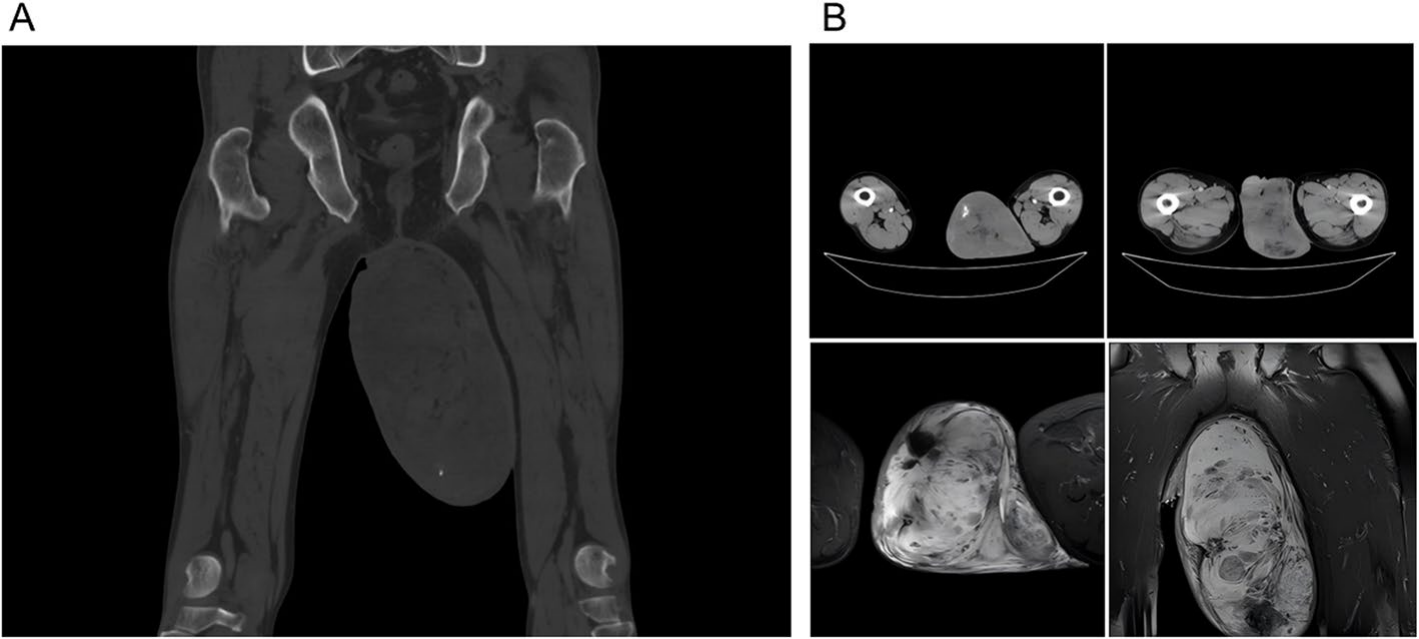
Images of the scrotal mass. **A**. Urologic computed tomography in the coronal view. B. Radiological images from different angles

Enhanced CT of the urinary tract indicated a left testicular space-occupying lesion that was potentially a germ cell tumor but other possibilities could not be excluded. Both kidneys were of normal size and morphology, no obvious abnormal density shadows were seen in the parenchyma of both kidneys, and no abnormal enhancement of bilateral renal parenchyma was seen in the enhancement CT scan. There was no dilatation of bilateral renal pelvis and ureter. The perirenal area was not different bilaterally. The bladder was not satisfactorily filled, with smooth walls and uniform density. The morphology, size and density of bilateral adrenal glands are not abnormal. size and density were not abnormal, no abnormal nodular shadows were seen, and no abnormal density was seen in the liver at the level seen. Left inguinal canal thickened, diameter about 24 mm, low density fat shadow can be seen inside. The volume of the left testicle increased significantly, ranging from 123 × 168 mm, with speckled calcification and low density fat shadow. Moreover, there was no apparent abnormality in the urinary tract, but the left inguinal canal was dilated (Fig. 1).

An MRI of the left scrotum included an abnormal signal in the scrotum in size of 12.6 cm * 9.4 cm, potentially indicating a space-occupying lesion from a germ cell tumor, but other possibilities could not be excluded. There were small patches of short T1 signal, mixed high signal in the T2 compression fat image, high and low mixed signal in the DWI high b-value sequence, and high signal in the ADC image. There were no obvious enlarged lymph node shadows in the inguinal area of the two sides.

No obvious abnormal signs were found in the right testicle under CT or MRI. Chest CT showed inflammation of the lower lobe of the left lung. Abdominal ultrasound showed other organs were normal.

### Surgical treatment

**Fig. 2:**
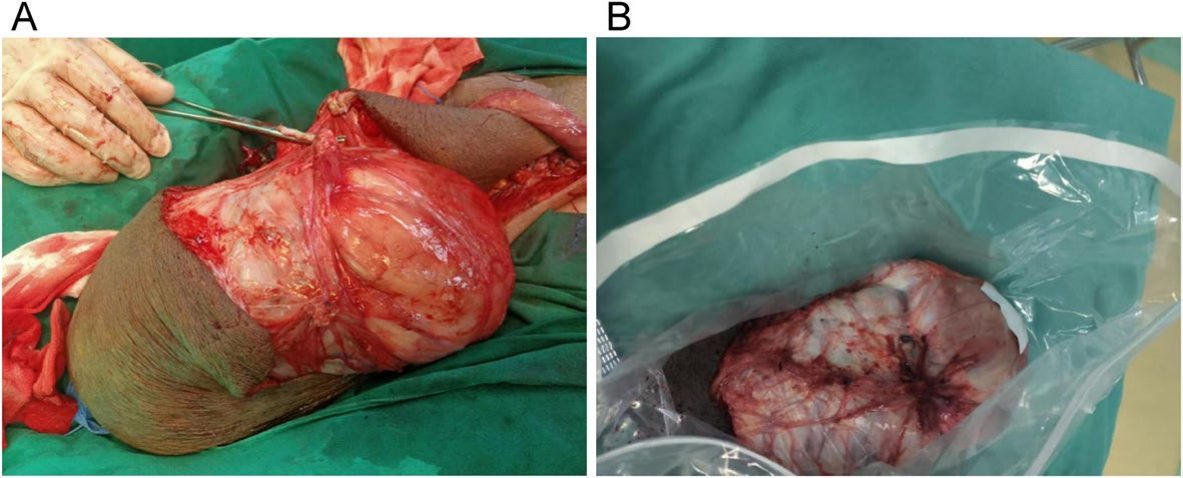
A. Intraoperative demonstration of a scrotal mass. **B**. The postoperative gross specimen

The left scrotal lesion was resected under lumbar anesthesia on 31 August 2023. Although a left testicular tumor resection was initially proposed, no abnormality of the left testis was identified intraoperatively. A 25-cm longitudinal incision was made in the left scrotum to incise the skin and membranes sequentially and to open the sheath membrane of the testis, which revealed normal morphology of the left testis and epididymis. A solid mass (25 × 15 cm) was adjacent to the sheath membrane and clearly demarcated from the testis. Complete excision of the mass was performed based on the pathologic examination. Excess scrotal skin was excised and hemostasis was achieved. A drainage tube was placed at the bottom of the left scrotum and connected to a negative-pressure drainage bulb to stabilize the testis. Instruments and dressings were accurately counted and the flesh membrane and skin were closed with 4–0 absorbable suture. The operation was successfully completed with an intraoperative blood loss of 50 ml. The surgical specimen appeared to be a single-skinned mass, measuring 255 × 163 × 90 mm, with an intact peritoneum (Fig. 2).

### Diagnosis

**Fig. 3:**
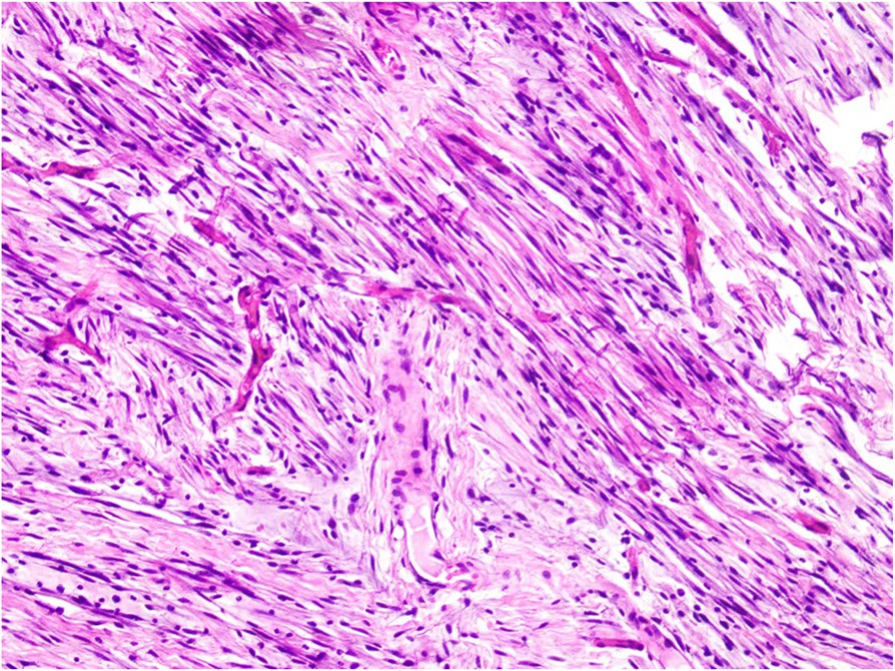
Pathologic manifestations of the scrotal masses indicating an angiomyofibroblastomic tumor of the male genital tract (20 × original magnification)

**Fig. 4:**
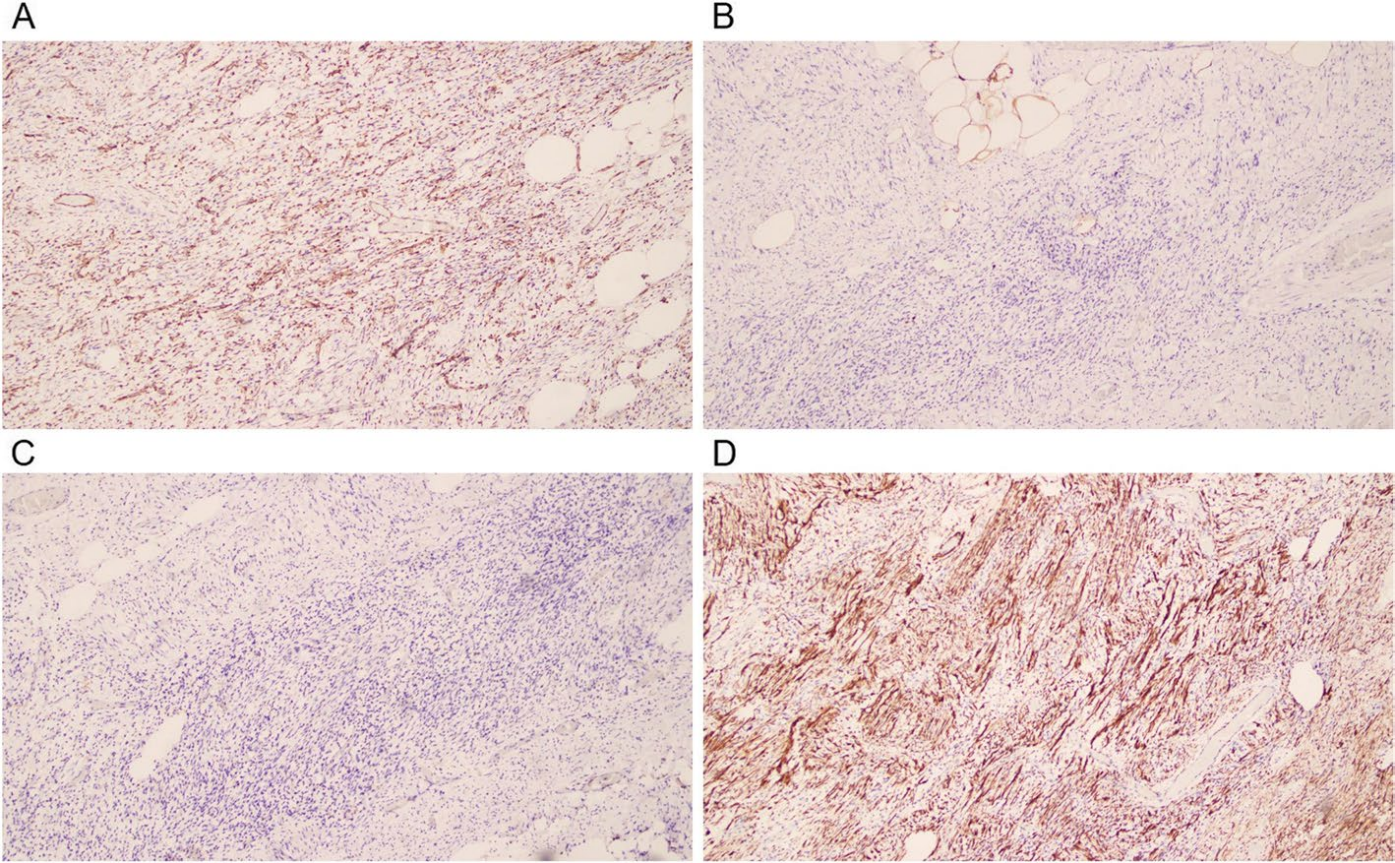
Immunohistochemistry results. **A**. CD31 (foci +) negative, indicating that the tumor cells did not originate from blood vessels; **B**. S-100 (–), positive, exclusive of neuronal origin and malignant melanoma; **C**. SALL-4 (–) negative, exclusive of reproductive source; **D**. desmin (+)

The pathologic examination revealed a single-skinned mass on gross appearance measuring 255 × 163 × 90 mm with an intact envelope and a gray-red/gray-yellow cut surface. The localized fish-like (left scrotal mass) tumor boundaries were clear and were mainly composed of spindle-shaped cells arranged in bundles. The cell morphology was mild with no heterogeneity or clear nuclear atypia. Scattered thin-walled blood vessels with small-to-moderately large lumens and fat components were observed in the tumor. The focal area showed necrosis. Based on morphology and immunohistochemical phenotypes, the patient was considered to have a male genital tract AMF tumor. The hematoxylin–eosin staining showed in Fig. 3. The immunohistochemistry results were as follows: CD31 (foci +) (Fig. 4A); S-100 (–) (Fig. 4B); SALL-4 (–) (Fig. 4C); desmin (+) (Fig. 4D); caldesmon (–); CD117 (mast cell +); CD34 (+); catenin-β (nuclear +); HMB45 (–); melan-A (–); P16 (partially +); smooth muscle actin (–); myogenin (–); OCT3/4 (–); SALL-4 (–); pancytokeratin (–); estrogen receptor (–); epithelial membrane antigen (small foci +); MyoD-1 (–); KI-67 (2% +); and progesterone receptor (–).

### Follow-up visits

The patient had regular follow-up evaluations after resection of the left scrotal lesion. One month postoperatively the patient was evaluated in the Outpatient Clinic at Hami City Center Hospital. The wound was healing well without any discomfort and no additional treatments were required. The patient is evaluated every 6 months postoperatively. He has reported no discomfort and superficial ultrasound and physical examinations have shown no abnormalities during the follow-up period.

### Literature review summary

Eight scrotal AMF cases [1–4, 6–9] reported in English are shown in Table 1. Other cases not in English or the published version that could not be accessed were not included into Table 1.

**Table 1:** Summary of scrotal AMF

Research	Age	Main complaints	Appearance feature	Imaging features	Histopathologic features	Treatment and diagnosis	Follow up
Owaidah et al., 2022 [1]	34	A large left testicular mass was noted without pain	Oval shape	MRI showed an indeterminate hypervascular left inguinal canal mass abutting the anterior aspect of the spermatic cord	Tumor markers, including beta-human chorionic gonadotropin, alpha-fetoprotein, and lactate dehydrogenase, were tested, all of which yielded negative results	Surgery; diagnosed as a para-testicular angiofibroma	Complete wound healing
Zeng et al., 2022 [2]	42	The mass remained the same size for 4 months and the patient felt slight tenderness in the left scrotum	6 cm × 5 cm mass	Imaging examinations showed that the mass had abundant vessels and displayed obvious progressive intensification on enhanced MRI	The tumor was rich in thin-walled, small blood vessels, and surrounded by hyperplastic spindle cells. Immunohistochemical testing showed that staining for muscle-specific actin (SMA) was positive and S100 was negative. Tumor cells were positive for desmin and CD34, and the Ki67 proliferation index was approximately 1%	The tumor was excised and diagnosed as an angiofibroma	Recovered well
Chih-Chen et al., 2016 [3]	89	An enlarged, painless, left scrotal mass for 1 year	Soft tissue-like mass, approximately 4.5 cm × 6.5 cm × 6.3 cm in size	Two solid scrotal masses were identified using scrotal ultrasound as well as two well-defined masses based on abdominal and pelvic computed tomography	Immunohistochemically, the tumor markers were CD34 (+), focally positive for S-100, actin (−), desmin (−), estrogen receptor (−), progesterone receptor (−), beta-catenin (−), CD99 (−), and B-cell lymphoma 2 (−)	The tumor was excised and diagnosed as a scrotal angiofibroma	Recovered and no tumor recurrence
Kass et al., 2019 [4]	64	A painless scrotal mass	Oval mass measuring 3.0 × 2.0 cm in size	A well-circumscribed, extra-testicular mass was present on ultrasonography	There was spindle cell proliferation with alternating hypocellular and hypercellular areas between the vascular channels. No mitoses were noted in these spindle cells. The spindle cells were strongly positive for CD34 and most of the tumor cell nuclei were typically and diagnostically positive for estrogen and progesterone. The SMA and S100 proteins were also positive	Diagnosed as a para-testicular AMF-like tumor that was treated surgically	Uneventful recovery from surgery
Ding et al., 2014 [6]	37	A painless mass in the left scrotum gradually increased in size	A mass ~ 4 × 5 cm in size	Scrotal ultrasonography showed a mass ~ 4 × 5 cm in size in the left scrotum that was not clearly differentiated from the testis and vascularity was observed inside and around the mass	Tumor markers, such as α-fetoprotein and human chorionic gonadotropin, had normal expression. The tumor cells stained positive for smooth muscle actin and negative for S-100, CD34, and actin	The pathologic diagnosis was a left scrotal AMF-like tumor. An inguinal orchiectomy was performed	No recurrences were detected over 7 years of follow up evaluations
Dave et al., 2023 [7]	64	Scrotal swelling with increased frequency of micturition	9.38 × 4.47 × 8.11 cm in size	Scrotal ultrasonography revealed a large, non-reducible, heterogeneous mass measuring 9.38 × 4.47 × 8.11 cm in the right scrotum above the testis	Microscopically, the lesion was composed of evenly distributed hypocellular and hypercellular areas. Small-to-medium capillary-sized hyalinized blood vessels were noted in the stroma. A pathologic examination showed features of a cellular AF/AMF-like tumor, which was positive for CD34 and negative for S-100 protein, smooth muscle actin, and desmin. Mib-1 labelling index was 2–4% in the highest proliferating areas. Mast cells were distributed amidst the neoplastic cells and were highlighted by C-Kit (CD117) immunohistochemistry	A diagnosis of an angiomyofibroblastoma (AMF)-like tumor or cellular AF was rendered. A para-testicular tumor excision was performed	N/A
Aytaç et al., 2012 [8]	40	A painless scrotal mass enlarged	Cut surface of the tumor measuring 6.5 × 4.5 × 2 cm	Doppler ultrasound of the scrotum revealed normal testes bilaterally and a 6 × 5 × 3 cm solid mass separate from the testis and epididymis. On magnetic resonance imaging, T1-weighted images showed hypointense and T2-weighted imaging showed hyperintense images	Spindle cells were separated by fine collagen fibers and abundant edematous background. The vascular component was prominent and haphazardly distributed throughout the tumor with irregularly thickened walls containing fibrinoid or hyalinized material. Perivascular arrangement of tumor cells with focal targeting or whirling pattern was also noted. Immunostaining of the tumor cells was strong for vimentin and smooth muscle actin (SMA) and were focally stained by desmin (Fig. 3). Additional stains were negative for cytokeratin, myogenin, S-100, estrogen, and progesterone receptor proteins. Stains for CD34 were also negative in the tumor cells but highlighted endothelial cells	The pathologic diagnosis was an angiomyofibroblastoma-like tumor. The tumor was excised	N/A
Lee et al., 2010 [9]	71	A chief complaint of a 1-year history of a mass in the right scrotum	The removed tumor was 13 × 10 × 6 cm in size and had an oval shape	Scrotal ultrasonography showed a giant mass, 12 cm in size, in the right scrotum	Tumor markers, including alpha-fetoprotein, beta-human chorionic gonadotropin, and lactate dehydrogenase, were all shown to be normal. Immunohistochemical staining showed negative findings for desmin, S-100, and CD34	Diagnosed as an angiomyofibroblastoma-like tumor that occurred in the scrotum. The tumor was excised	Recovered well with no local recurrences

Scrotal AMFs occurred in patients ≥ 30 years of age. Patients often detect an oval-shaped mass in the scrotum without other clinical symptoms. MRI, CT, and ultrasound can be used to determine the indeterminate hypervascular in the abnormal mass. Sometimes the tumor makers were negative but tumor markers were most often positive. All patients underwent surgical treatment and recovered fully.

## Discussion

The clinical symptoms and physical examination findings of patients with scrotal AMF lack specificity, making a definitive diagnosis before surgery challenging, as shown in Table 1. Accurate diagnosis mainly relies on pathologic and immunohistochemistry findings and imaging can only be used as a reference [1–4, 6–9]. The morphology of scrotal AMF is specific with clear boundaries distinguishing scrotal AMF from surrounding tissues and a somewhat round shape [1, 4, 9], usually with a diameter < 5.0 cm. The largest reported diameter of scrotal AMF is 13 × 10 × 6 cm [9]. The current case reported a diameter of 25 × 15 cm in size, which is rarely reported. The patient lived in remote area in the mountains, and he had knowledge and little attention about his own health problems. That may be why the tumor grew to so big until he came to hospital for treatment. However, AMF occurring in women may be larger. In fact, the largest AMF report in women is 34 cm [10].

The tumor is elastic and soft, spongy or mucinous, and white or yellow–brown in color but lacks a fibrous or pseudo-coated surface and does not undergo hemorrhage or necrosis. Microscopically, the tumor is enriched with thin-walled blood vessels and scattered myofibroblasts, most of which are aggregated around the vessels in sparse and dense distributions [11, 12]. The myofibroblasts are spindle-shaped or ovoid and slender or obese, with a moderate amount of slightly acidic cytoplasm. The nucleus is oval with fine chromatin and inconspicuous nucleoli, occasionally showing nuclear atypia. Some cells are ovoid, with eosinophilic translucent cytoplasm, resembling those in plasma cell–like salivary gland mixed tumors [11, 12]. The arrangement of tumor cells in sparsely cellular areas is consistent with the direction of collagen fibers [2, 7, 8]. The clinical features, histologic structure, and immunophenotype of the patient in this case report were consistent with the literature [2, 7, 8, 11, 12].

MRI, CT, and ultrasound are often used to helped diagnose scrotal AMF in clinical practice. The current case report showed that an abnormal signal in the scrotum in size of 12.6 cm * 9.4 cm with a clear boundary under MRI of the left scrotum [1, 4, 9]. Previous reports showed that germ cell tumors under MRI showed multiple nodules, and the number of fibrous and vascular compartments varied, and the thickness was uneven [13]. The images and clinical differences were summarized in Table 2, which should be focused in clinical practice [1–9, 13]. AMF is often benign [1–9], and germ cell tumor is often malignant [13]. However, the accurate diagnosis of scrotal AMF still need the pathological findings to support.

**Table 2:** The CT differences between AMF and germ cell tumors

Identify the key points	AMF	Germ cell tumors
The CT scan characteristics	Most tumors often appear as well-defined soft tissue masses with uniform or uneven density, and the enhancement scans show light to moderate enhancement, and the degree of enhancement is related to the vascular component within the tumor. Low-density areas were seen in some tumors, with mucus degeneration or cystic change	CT findings of different types of germ cell tumors. Teratoma shows fat, calcification, soft tissue and characteristic fat density; seminoma is a uniform soft tissue density mass; endodermal sinus tumor is often a large mass, accompanied by necrosis and hemorrhage
The MRI scan characteristics	It showed more equal signals on T 1 WI and high signals on T 2 WI, and the signal intensity was related to the proportion of cells, collagen fibers and mucus components within the tumor. The enhancement was mild early in the enhanced scan and increased in the delayed phase. If there is bleeding in the tumor, each sequence of signal performance is related to the bleeding time	Teratoma showed mixed signals in the MRI sequence, adipose components showed high signal in T 1 WI and T 2 WI, and the signal decreased; seminoma T 1 WI showed equal signal, T 2 WI showed slightly higher signal, and enhanced enhancement was less than that of normal testicular tissue; endoderoma T 1 WI and T 2 WI showed high signal, and uneven enhancement
Pathway of growth	It usually grows expansively with clear boundaries and a clear demarcation from surrounding tissues, with less invasion of surrounding tissues	The growth mode is diverse, some show expansion growth, some can show invasive growth, such as malignant germ cell tumors can invade the surrounding tissues and structures, and the surrounding tissues are unclear demarcation
D reg	It is commonly found in the female vulva and vagina, but can also occur in other pelvic sites	Good hair in the pineal area, supraslar area, testis, ovary and other gonads and body midline, such as mediastinal, sacral tail, etc

When combined with pathologic findings, an AMF diagnosis can be established [1–9]. The most important clinical implication for urologic practice is the avoidance of overtreatment. AMF is a benign tumor, and correct diagnosis avoids unnecessary orchiectomy or radical surgery. Surgical treatment is the optimal choice when an abnormal mass is confirmed in the scrotum [1–9]. However, negative tumor markers are not consistent with a lesion. This situation highlights the difficulty in diagnosing scrotal AMF. Therefore, further in-depth studies with a focus on the pathogenesis of AMF and optimal therapeutic strategies are needed. Because of the low prevalence of scrotal AMF, multicenter collaboration involving imaging, urology, and pathology departments will help distinguish AMF and enable the patient to undergo treatment as early as possible. For the initial evaluation and management of patients presenting with scrotal masses of unknown cause, exhaustive specialty workup is paramount, and AFP CT MRI remains an important adjunctive test for identifying AMF, germ cell tumors, and intraoperative re-examination is critical to avoid misdiagnosis and misdiagnosis. As the age of AI approaches, AI-assisted image analysis of such research areas is particularly critical to improving our understanding of scrotal AMF. To achieve better therapeutic outcomes, treatment strategies for AMF should be based on rigorous imaging evaluation and pathologic analysis, as well as the clinical characteristics and individual differences.

No reports exist of patients with recurrent or advanced disease [1–9, 11, 12]. The patient in this case report recovered fully. Further follow-up with this case report is needed.

Scrotal AMF is a rare tumor that has an oval shape in the scrotum the size of a chicken egg. Patients often feel the mass but are asymptomatic. Imaging, pathologic findings, and tumor biomarkers can help diagnose and treat the patient. Surgery is the optimal option for patients and no recurrences have been reported in patients with scrotal AMF. However, due to the low incidence of AMF, few in-depth studies have been conducted. Research on scrotal AMF is still in the early stage and further exploration of the etiology, pathogenesis, and therapeutic strategies is needed.

## Supplementary Information

Supplementary Material 1. 

## Data Availability

The datasets used and/or analysed during the current study are available from the corresponding author on reasonable request.

## Abbreviations

AMF: Angiomyofibroblastoma
CT: Computed tomography
MRI: Magnetic resonance imaging
BMI: Body mass index
